# Psychosocial characteristics of potential and actual living kidney donors

**DOI:** 10.1186/s12882-023-03375-z

**Published:** 2024-01-24

**Authors:** Liza K. Cholin, Everly F. Ramos, Jordana Yahr, Jesse D. Schold, Emilio D. Poggio, Christina L. Delvalle, Anne M. Huml

**Affiliations:** 1 Department of Nephrology, The Ohio State University Wexner Medical Center, 300 W 10Th Ave, Columbus, OH #1150 USA; 2 Department of Internal Medicine, Cleveland Clinic, Cleveland, USA; 3 Department of Surgery and Transplant, University of CO Anschutz Medical Campus, Aurora, CO, USA; 4 Department of Kidney Medicine, Cleveland Clinic, Cleveland, OH USA; 5 Department of Transplantation, Cleveland Clinic, Cleveland, OH USA

**Keywords:** Living donor, Psychoscocial risk, Kidney donor evaluation

## Abstract

The psychosocial assessment is an essential component of the living kidney donor (LKD) evaluation. However, it remains uncertain how specific psychosocial factors impact LKD eligibility. We performed a retrospective chart review of LKD candidates who initiated the evaluation process and who had completed a required, in-person licensed social work (LSW) visit. LSW notes were reviewed for frequency of psychosocial factors that may impact the success of LKD candidate approval by the selection committee. 325 LKD candidates were included in the study: 104 not-approved and 221 approved. Not-approved LKD candidates were more likely to receive a negative family reaction to wanting to donate than approved LKD candidates (8.7% vs 1.4%, *p* < 0.01). On multivariate analysis, Black race, history of psychiatric illness, highest level of education being high school, and high psychosocial risk score assignment were all associated with a lower odds ratio of being approved. The majority of not-approved LKD candidates were disqualified for medical reasons (*N* = 76, 73.1%). In conclusion, psychosocial factors impact donation even after LKD candidates make it to an in-person evaluation.

## Introduction

Living kidney donors (LKD) are in a crucial position to help the growing demand of kidneys in the United States. An important part of a donor’s evaluation is the psychosocial assessment. The goal of this assessment is to measure decision making ability, to evaluate for possible undue pressure to donate or coercion, and to identify any psychosocial risk factors that may complicate the recovery process and long-term outcomes of the potential donor [1, 2]. Despite general agreement that the psychosocial assessment is a necessary component of the LKD evaluation, there is little consensus on the best method to implement it [3–5]. There is significant heterogeneity among transplant center practices regarding assessment and acceptance thresholds of psychosocial risk among LKD candidates [6]. As a result, some centers have implemented psychosocial assessment tools to standardize psychosocial risk and better anticipate post-donation needs [7, 8].

There are 9 general categories that have been accepted as part of the LKD psychosocial evaluation and they include relationship to the recipient, motivation to donate, knowledge of the donation process, donor’s feelings about donation, psychiatric history, substance use, post-donation plan, social support, and overall stability in life [8]. Certain psychosocial factors are thought to increase the risk for post-donation adverse outcomes; these factors include unrelated donor-recipient relationship, history of psychiatric disorder, and poor socioeconomic status [9, 10]. However, there are other factors that are standard to the psychosocial assessment that are less clear in terms of how they impact donation. This study aimed to assess all psychosocial factors that are evaluated at a large transplant center, and to determine how each of these psychosocial factors impact a LKD candidate’s likelihood to be approved by the selection committee.

## Methods

### Study design

A retrospective chart review was performed on LKD candidates who initiated the donation process at a large transplantation center between December 23, 2016 and December 31, 2019. Prior to being seen in-person, LKD candidates were required to complete a screening questionnaire and preliminary immunologic compatibility testing. LKD candidates were required to be between 18 and 75 years of age, have a BMI ≤ 35 kg/m^2^, and be willing to accept a blood transfusion. In addition, candidates were only considered if they showed no evidence of diabetes, uncontrolled hypertension, active malignancy, acute infection, HIV infection, or active hepatitis B or C. After reviewing these results, the transplant center invited suitable donor candidates for further evaluation in clinic. ABO incompatible LKD donor candidates interested in paired donation were allowed to continue in their evaluation. A mandatory component of the clinic evaluation included psychosocial assessment by a licensed social worker (LSW). For this study, we included only LKD candidates who had completed a LSW evaluation.

The LSW evaluation was an in person, single day assessment utilizing a standardized note template. Topics on the template included relationship to recipient, prior altruistic behavior, motivation to donate, understanding of recipient’s health status, decision- making ability, substance use, mental health history, living situation, education, employment status, and plans for donation and recovery. LSWs were required to answer all of the questions on the template. At the conclusion of the evaluation, LSWs assigned a low, moderate, or high psychosocial risk score to the LKD candidate. Risk assignments were made based on the LSW’s overall impression, rather than a numerical scoring system.

LKD candidates that completed LSW evaluation were further subdivided according to approval decision by the selection committee. Not-approved LKD candidates were compared to approved LKD candidates to determine whether there were any differences in their respective psychosocial assessments, which may have impacted their ability to complete donation. This study was deemed exempt by the Institutional Review Board.

### Variables

The electronic health record was used to access LKD candidates’ psychosocial evaluations. Responses to the questions listed in the LSW template were collected for all LKD candidates evaluated. In addition, the LSW psychosocial risk assignment was obtained, as it contributed to the selection committee decision. Reasons for higher psychosocial risk score and selection committee denial were collected, when applicable.

### Statistical analysis

A descriptive analysis was performed on the psychosocial factors collected. The mean or median was calculated for continuous variables, while frequency and percentages were reported for categorical variables. A logistic regression model was created to determine the overall effect each psychosocial factor had on donor approval. A small portion of the variables were found to be collinear when running the regression model, thus, they were not included in the multivariate analysis.

Risk factors were tallied for each LKD candidate. A T-test was used to compare mean risk factor count between approved and not-approved groups. All analyses were conducted using SAS-JMP version 15, Cary, NC.

## Results

### Psychosocial characteristics

Table 1 lists the responses from the psychosocial evaluation for approved and not-approved LKD candidates. Approved LKD candidates were more often younger (age 42 vs 47, *p* = 0.25), female (65.2% vs 60.6%, *p* = 0.42), and white (91.4% vs 74.0%, *p* < 0.01). A blood relative was the most common donor-recipient relationship for both groups, 48.0% and 51.0% (*p* = 0.59). The approved group was less likely to report substance use or a psychiatric illness than the not-approved group, 24.4% vs 37.5% (*p* = 0.02) and 32.6% vs 42.3% (*p* = 0.09), respectively. More approved LKD candidates completed college than not-approved LKD candidates, 67.4% vs 51.0% (*p* = 0.01). Approved LKD candidates were also more likely to be employed (86.0% vs 80.8%, *p* = 0.47) and married/in-a-partnership (72.0% vs 59.6%, *p* = 0.08).

**Table 1:** Psychosocial Factors among Not-Approved and Approved LKD Candidates

	**Not Approved (*N* = 104)**	**Approved (*N *= 221)**
**Age, median (25, 75**^**th**^** quantile)**	46.5 (34.3, 56)	42 (32, 53)
**BMI, median (25, 75**^**th**^** quantile)**	26.6 (23.7, 29.6)	25.8 (23.3, 29.8)
**Gender, N (%)**		
Male	41 (39.4)	77 (34.8)
Female	63 (60.6)	144 (65.2)
**Race, N (%)**		
White	77 (74.0)	202 (91.4)
Black	21 (20.2)	10 (4.5)
Other	6 (5.8)	9 (4.1)
**Relationship to recipient, N (%)**		
Blood relative	53 (51.0)	106 (48.0)
Spouse/significant other	11 (10.6)	29 (13.1)
Step-family or in-law	9 (8.7)	12 (5.4)
Not related	31 (29.8)	74 (33.5)
**Previous blood donor, N (%)**	68 (65.4)	163 (74.1)
**Registered as organ donor on I.D., N (%)**	73 (70.2)	169 (77.9)
**Knows cause of recipient’s CKD, N (%)**	69 (66.4)	158 (71.5)
**Gets regular physicals, N (%)**	75 (72.1)	158 (71.8)
**Current substance use, N (%)**	39 (37.5)	54 (24.4)
**Type of substance used, N (%)**		
Alcohol	9 (8.7)	18 (8.1)
Tobacco	11 (10.6)	17 (7.7)
Marijuana	2 (1.9)	8 (3.6)
Multiple substances	17 (16.4)	11 (5.0)
No use	65 (62.5)	167 (75.6)
**Remote substance use, N (%)**	64 (61.5)	98 (44.3)
**Diagnosis of psychiatric disorder, N (%)**	44 (42.3)	72 (32.6)
**Currently on psych medication, N (%)**	41 (26.8)	34 (18.3)
**Owns home, N (%)**	77 (74.0)	193 (87.7)
**Drives car, N (%)**	100 (97.1)	219 (99.1)
**Living with dependent, N (%)**	42 (40.4)	107 (48.4)
**Owns pet, N (%)**	64 (62.1)	154 (71.3)
**Share of household income, N (%)**		
Split	62 (59.6)	141 (63.8)
Minimal	14 (13.5)	26 (11.8)
Majority	28 (26.9)	54 (24.4)
**Share of household tasks, N (%)**		
Split	67 (64.4)	153 (69.2)
Minimal	10 (9.6)	19 (8.6)
Majority	27 (26.0)	49 (22.2)
**Has access to computer and is computer literate, N (%)**	97 (94.2)	218 (99.1)
**Highest level of education completed, N (%)**		
High school	49 (47.1)	62 (28.1)
Trade school	2 (1.9)	10 (4.5)
Undergraduate	34 (32.7)	93 (42.1)
Graduate	19 (18.3)	56 (25.3)
**Currently employed, N (%)**		
Yes	84 (80.8)	190 (86.0)
No	11 (10.6)	18 (8.1)
Retired	9 (8.7)	13 (5.9)
**Has paid leave, N (%)**		
Yes	56 (54.4)	134 (61.2)
No	38 (36.9)	72 (32.9)
Retired	9 (8.7)	13 (5.9)
**Has health insurance, N (%)**	96 (92.3)	208 (94.1)
**Marital status, N (%)**		
Married/in a partnership	62 (59.6)	159 (72.0)
Widowed/divorced	15 (14.4)	22 (10.0)
Single	27 (26.0)	40 (18.0)
**Family reaction to interest in donation, N (%)**		
Positive with some reservations	31 (29.8)	79 (35.9)
Negative	9 (8.7)	3 (1.4)
Very positive	64 (61.5)	138 (62.7)
**Requires social support from outside the home, N (%)**	58 (56.3)	101 (46.1)
**Has religious or spiritual support, N (%)**	70 (68.0)	156 (70.6)
**Has financial support, N (%)**	99 (95.2)	215 (98.2)
**Has advanced directive, N (%)**	16 (15.4)	43 (19.6)
**Assigned psychosocial risk score, N (%)**		
Low risk	56 (53.9)	165 (74.7)
Moderate risk	35 (33.7)	55 (24.9)
High risk	13 (12.5)	1 (0.5)

The following categories had missing responses: previous blood donor (1), registered as organ donor on I.D. (4), gets regular physicals (1), owns home (1), drives car (1), owns pet (6), has access to computer (2), has paid leave (3), family reaction to donating (1), requires social support from outside home (3), religious or spiritual support (1), financial support (2), has advance directive (1)

**Fig. 1:**
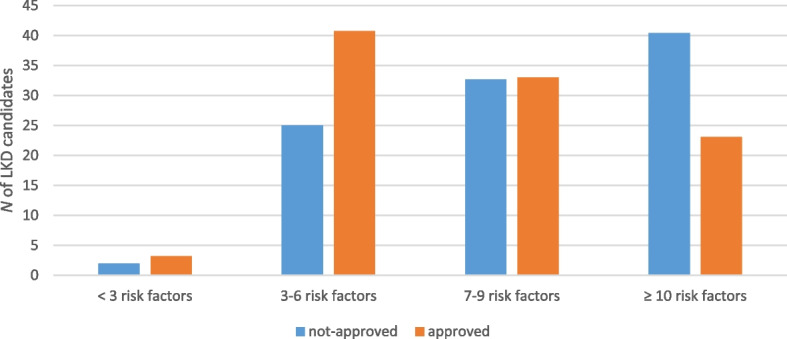
Psychosocial Risk Factor Count

There was a high rate of altruistic behavior amongst both groups, with a majority of LKD candidates reporting either previous blood donation (65–74%) or being registered as an organ donor on their I.D. (70–78%, Table 1). The rate of potential donors that knew the recipient’s cause of CKD was similar between approved and not-approved groups, 71.5% vs 66.4% (*p* = 0.32), respectively. The rates of approved LKD candidates that got regular physicals was also similar to the rate of not-approved LKD candidates, 71.8% and 72.1% (*p* = 0.96). Significantly more approved LKD candidates owned a home than not-approved LKD candidates, 87.7% vs 74.0%, *p* < 0.01. There was no statistical difference between the two groups with regard to transportation, in-home dependents, share of household income, share of household tasks, religious/spiritual support, or financial support. There was a trend towards more not-approved LKD candidates requiring social support from outside the home than approved candidates, 56.3% vs 46.1% (*p* = 0.09). Not-approved LKD candidates were also more likely to receive a negative family reaction to wanting to donate than approved LKD candidates, 8.7% vs 1.4% (*p* < 0.01). Figure 1 illustrates the psychosocial risk factor count between approved and not-approved LKD candidates. Not-approved LKD candidates had a statistically higher total risk factor count than approved candidates (8.9 vs 7.2, *p* < 0.01).

### LSW Impression

46.2% of not-approved LKD candidates received a moderate or high psychosocial risk score compared to 25.3% of approved LKD candidates (Table 1; *p* < 0.01). Substance use (31.7%) and mental health concerns (28.9%) were the two most common reasons for higher risk score assignment amongst all LKD candidates (Table 2). Other less common reasons included financial concerns, lack of social support/post-donation plan, and second thoughts about donation.

**Table 2:** Reason for Moderate or High Risk Social Work Score

	**Overall (*N* = 104)**	**Not Approved (*N* = 48)**	**Approved (*N* = 56)**
Substance use	33 (31.7)	16 (33.3)	17 (30.4)
Mental health concern	30 (28.9)	14 (29.2)	16 (28.6)
Substance use and mental health concern	6 (5.8)	3 (6.3)	3 (5.4)
Financial concern	8 (7.7)	3 (6.3)	5 (8.9)
Lack of social support/post-donation plan	4 (3.9)	1 (2.1)	3 (5.4)
Second thoughts about donation	5 (4.8)	3 (6.3)	2 (3.6)
Multiple reasons/other	18 (17.3)	8 (16.7)	10 (17.9)

### Selection committee decision

Reason for selection committee denial was determined for not-approved LKD candidates (Table 3). LKD candidates were most often disqualified for medical reasons (*N* = 76, 73.1%). 10 (9.6%) were not approved for psychosocial reasons, and another 9 (8.7%) were declined for a combination of medical and psychosocial reasons. 9 (8.7%) LKD candidates were lost to follow up after completing LSW evaluation, but before selection committee review.

**Table 3:** Reason for Denial

	**Overall Not Approved Group (*N* = 104)**	**Not Approved Group with Moderate or High Risk SW Score (*N *= 48)**
Medical reason only	76 (73.1)	24 (50.0)
Psychosocial reason only	10 (9.6)	10 (20.8)
Medical and psychosocial reason	9 (8.7)	7 (14.6)
Lost to follow up	9 (8.7)	7 (14.6)

^*^14 potential donors were eliminated from the study because their recipient wasn’t listed or they specifically stated they wanted to stop the evaluation before a committee decision could be made. However, a few “not approved” donors (*N* = 9) were included in the study who did not have a clear reason for drop out

### Multivariate analysis

A logistic regression model was performed on most of the psychosocial factors assessed (Table 4). A few of the variables had a collinear effect, and therefore, were not included in the model (see “N/A” under multivariate analysis column). Black race, history of psychiatric illness, highest level of education completed being high school, and a high psychosocial risk score were all associated with a lower odds ratio of being approved by the selection committee.

**Table 4:** Univariate and Multivariate Analysis among Not-Approved and Approved LKD Candidates

	**Univariate Analysis, *p*-value**	**Multivariate Analysis, OR (95% CI)**
**Age**	0.25	1.05 (1.01, 1.09)
**BMI**	0.68	N/A
**Gender**	0.42	
Male		Ref
Female		1.51 (0.77, 2.98)
**Race**	< 0.01	
White		Ref
Black		0.10 (0.03, 0.33)
Other		0.30 (0.07, 1.34)
**Relationship to recipient**	0.59	
Blood relative		Ref
Spouse/significant other		1.25 (0.42, 3.69)
Step-family or in-law		0.65 (0.18, 2.33)
Not related		1.18 (0.55, 2.55)
**Previous blood donor**	0.11	1.08 (0.54, 2.19)
**Registered as organ donor on I.D**	0.13	0.88 (0.41, 1.86)
**Knows cause of recipient’s CKD**	0.32	1.48 (0.73, 3.0)
**Gets regular physicals**	0.96	0.98 (0.45, 2.13)
**Current substance use**	0.02	0.85 (0.35, 2.02)
**Type of substance used**	0.01	
Alcohol		N/A
Tobacco		N/A
Marijuana		N/A
Multiple substances		N/A
No use		N/A
**Remote substance use**	< 0.01	0.82 (0.38, 1.76)
**Diagnosis of psychiatric disorder**	0.09	0.39 (0.18, 0.83)
**Currently on psych medication**	0.01	N/A
**Owns home**	< 0.01	N/A
**Drives car**	0.17	1.78 (0.14, 21.9)
**Living with dependent**	0.18	1.07 (0.52, 2.21)
**Owns pet**	0.10	0.78 (0.37, 1.65)
**Share of household income**	0.76	
Split		Ref
Minimal		1.87 (0.49, 7.20)
Majority		1.39 (0.51, 3.76)
**Share of household tasks**	0.68	
Split		Ref
Minimal		0.57 (0.18, 1.77)
Majority		1.12 (0.43, 2.94)
**Has access to computer and is computer literate**	0.01	N/A
**Highest level of education completed**	0.01	
High school		0.32 (0.15, 0.66)
Trade school		5.07 (0.49, 52.5)
Undergraduate		Ref
Graduate		0.83 (0.36, 1.91)
**Currently employed**	0.47	
Yes		Ref
No		0.78 (0.17, 3.55)
Retired		0.72 (0.20, 2.61)
**Has paid leave**	0.43	
Yes		N/A
No		N/A
Retired		N/A
**Has health insurance**	0.54	0.41 (0.10, 1.70)
**Marital status**	0.08	
Married/in a partnership		Ref
Widowed/divorced		0.55 (0.17, 1.73)
Single		0.40 (0.13, 1.24)
**Family reaction to interest in donation**	< 0.01	
Positive with some reservations		Ref
Negative		0.23 (0.04, 1.48)
Very positive		1.11 (0.57, 2.15)
**Requires social support from outside the home**	0.09	0.82 (0.38, 1.76)
**Has religious or spiritual support**	0.63	1.30 (0.62, 2.71)
**Has financial support**	0.13	2.30 (0.37, 14.2)
**Has advanced directive**	0.36	2.10 (0.90, 4.89)
**Assigned psychosocial risk score**	< 0.01	
Low risk		Ref
Moderate risk		0.74 (0.35, 1.57)
High risk		0.01 (< 0.01, 0.12)

## Discussion

LKD candidates in our study had similar demographics to LKDs across the nation; majority of the candidates were white, female, and related to the recipient [11]. Compared to not-approved LKD candidates, approved LKD candidates were less likely to report substance use, psychiatric illness, unemployment status, or require social support outside the home. Approved candidates were also more likely to be married/in-a-partnership, own a home, have a higher level of education, and have a positive family reaction to wanting to donate. Despite these differences, a minority of LKD candidates were disqualified for psychosocial reasons, with a greater portion of candidates being eliminated for medical reasons.

There is limited research on the psychosocial assessment of LKDs. Most of the literature on this topic has focused on the assessment variability between transplant centers, and on general post-donation outcomes. LKDs, overall, have good psychosocial outcomes with approximately 95% of donors reporting a good to excellent donation experience [12]. Additionally, only a small number of donors (4–11%) experience post-donation depressive symptoms [13, 14]. Predictive factors associated with worse psychosocial outcomes include younger age at donation, higher financial burden, and recipient graft failure [13–18]. To the best of our knowledge, our study is the first to look at how psychosocial factors influence the donor evaluation process.

The results from this study suggest that psychosocial factors impact approval by the LKD selection committee, with approximately 17% of LKD denials occurring, in part, due to psychosocial concerns. A recent survey of US transplant programs showed high variability in psychosocial issues that were considered a contraindication to donation [6]. Given the large variability in interpreting psychosocial risks between transplant centers, it is crucial to standardize the psychosocial assessment process. One potential method to increase uniformity across transplant programs is the utilization of a standardized assessment tool as part of the psychosocial evaluation. Several programs have implemented such tools with promising results [7, 8]. In particular, the living donor assessment tool produced by Kook and colleagues not only showed good reliability between raters, but was also able to predict outcomes of the psychosocial evaluation across multiple transplant centers.

The single center nature of our trial limits its generalizability to other transplant centers. However, it does offer the advantage of streamlining the psychosocial evaluation process. Despite this, inter-rater variability may have biased some of the study’s findings. In addition, the LKD candidates who were assessed were highly selected in order to obtain granular psychosocial information from the LSW evaluation. By excluding LKD candidates that failed initial screening, additional psychosocial variables that impact donation may have been missed. The findings from our study were also limited by the relatively small sample size and number of variables considered. Future work, using propensity matching, is needed to better understand how individual psychosocial factors impact LKD candidate approval. Improved tracking of post-donation outcomes is also important to enhance our knowledge on the influence psychosocial factors have on living donors. Currently, the required living donor follow up form by the Organ Procurement and Transplantation Network is limited to clinical and functional outcomes without psycho-social related items [19].

The psychosocial assessment remains a crucial part in the living donation process. The results from this study demonstrated that, cumulatively, psychosocial factors have considerable impact on LKD candidate approval by the selection committee. However, data is lacking on which specific factors put a LKD at highest risk for adverse post-donation outcomes. Out of an abundance of caution, it is possible that some transplant centers may be eliminating suitable donors due to perceived higher psychosocial risk. More research is still needed to determine donor safety in those candidates with varied psychosocial risk factors.

## Data Availability

The datasets generated and/or analyzed during the current study are not publicly available due privacy restrictions but are available from the corresponding author on reasonable request.

## Abbreviations

LKD: Living kidney donor
LSW: Licensed social worker
